# Could the preoperative urethral curve be used to predict immediate urinary continence following Retzius-sparing robot-assisted radical prostatectomy? A retrospective multicenter study

**DOI:** 10.1590/S1677-5538.IBJU.2026.0376

**Published:** 2026-08-05

**Authors:** Bo-Han Lin, Yang Chen, Zhi-Bin Ke, Chen-Hao Wang, Ye-Hui Chen, Xiao-Li Yan, Jian-Zhou Liu, Jun-Jie Fan, Qi-Jun Xu, Shao-Bin Zhuang, Zhong-Hua Zhu, Shao-Hao Chen, Qing-Shui Zheng, Xue-Yi Xue, Jun-Long Zhuang, Yong Wei, Ning Xu

**Affiliations:** 1 Fujian Medical University The First Affiliated Hospital Department of Urology Fuzhou China Department of Urology, Urology Research Institute, The First Affiliated Hospital, Fujian Medical University, Fuzhou, China;; 2 Fujian Medical University Binhai Campus of the First Affiliated Hospital Department of Urology Fuzhou China Department of Urology, National Regional Medical Center, Binhai Campus of the First Affiliated Hospital, Fujian Medical University, Fuzhou, China;; 3 Shandong University Cheeloo College of Medicine Department of Urology Jinan China Department of Urology, Qilu Hospital, Cheeloo College of Medicine, Shandong University, Jinan, China;; 4 Baoji Central Hospital Department of Urology Baoji China Department of Urology, Baoji Central Hospital, Baoji, China;; 5 Fujian Medical University The First Affiliated Hospital Department of Radiology Fuzhou China Department of Radiology, The First Affiliated Hospital, Fujian Medical University, Fuzhou, China; 6 Fujian Medical University Binhai Campus of the First Affiliated Hospital Department of Radiology Fuzhou China Department of Radiology, National Regional Medical Center, Binhai Campus of the First Affiliated Hospital, Fujian Medical University, Fuzhou, China; 7 Fujian Medical University First Affiliated Hospital Fujian Key Laboratory of Tumor Immunotherapy Fuzhou China Fujian Key Laboratory of Tumor Immunotherapy, the First Affiliated Hospital, Fujian Medical University, Fuzhou, China; 8 Nanjing University Affiliated Hospital of Medical School Department of Urology Nanjing China Department of Urology, Nanjing Drum Tower Hospital, Affiliated Hospital of Medical School, Nanjing University, Nanjing, China; 9 Nanjing University Institute of Urology Nanjing China Institute of Urology, Nanjing University, Nanjing, China

**Keywords:** Prostatic Neoplasms, Prostatectomy, Urinary Incontinence

## Abstract

**Purpose:**

Immediate urinary continence (UC) recovery following Retzius-sparing robot-assisted radical prostatectomy (RS-RARP) remains highly variable, highlighting the need for reliable preoperative prediction. We aimed to develop and validate models to identify patients likely to achieve immediate UC recovery following RS-RARP.

**Materials and Methods:**

A total of 580 prostate cancer patients who underwent RS-RARP from four medical centers were assigned to a training set (n=348), an internal validation set (n=103) and an external validation set (n=129). Independent predictors were identified through univariate analysis and LASSO regression. A nomogram was constructed using multivariate logistic regression. Its performance was evaluated with receiver operating characteristic (ROC) curve, calibration curves, and decision curve analysis.

**Results:**

Immediate UC recovery was observed in 84.5% (294/348) of patients in the training cohort, 80.6% (83/103) in the internal validation cohort, and 81.4% (105/129) in the external validation cohort, respectively. Multivariate analysis identified membranous urethral length (MUL) (OR=1.23, P=0.029) and urethral curvature (OR=2.84, P<0.001) as independent predictors, while prostate volume (PV) (OR=0.84, P<0.001) as a protective factor. The nomogram integrating MUL, PV, and urethral curvature demonstrated superior predictive accuracy, with an AUC of 0.87 (95% CI, 0.83-0.91) in the training cohort. The bootstrap-corrected calibration slope was 0.96, and the Brier score was 0.08. Calibration curves and decision curve analysis confirmed the predictive accuracy and clinical utility of the nomogram.

**Conclusions:**

Our study introduces a novel quantitative method for assessing urethral curvature. The mpMRI-based model, integrating urethral curvature and prostate spatial configuration, offers enhanced predictive accuracy for postoperative immediate UC recovery.

## INTRODUCTION

Prostate cancer (PCa) is the second most common malignancy among men globally (1), and its incidence in China has increased rapidly due to expanded screening (2). Radical prostatectomy (RP) is one of the standard treatment options for localized and locally advanced PCa (3-5). However, urinary incontinence, the most common postoperative complication, significantly impacts quality of life (5). Although long-term urinary continence remains an important functional endpoint after radical prostatectomy, immediate UC recovery has attracted increasing attention because it directly reflects the effectiveness of anatomical preservation during surgery and has a substantial impact on early postoperative quality of life and functional recovery (6-8). Therefore, identifying patients who are likely to achieve immediate UC before surgery may facilitate individualized counseling and perioperative management.

Advancements in minimally invasive surgery have made robot-assisted radical prostatectomy (RARP) the predominant approach (9-11). In 2010, Galfano et al. (12) introduced Retzius-sparing RARP (RS-RARP), which offers a posterior approach that preserves the neurovascular bundle (NVB) and improves early urinary continence (UC) outcomes compared to the traditional anterior approach (12, 13). Nevertheless, the incidence of immediate UC recovery after RS-RARP ranges from 38% to 80% (14, 15), with some patients still failing to achieve immediate UC postoperatively. Therefore, identifying reliable preoperative predictors of immediate UC recovery following RS-RARP is clinically critical for risk stratification and surgical planning.

In recent years, preoperative multiparametric magnetic resonance imaging (mpMRI) has gained attention for predicting postoperative UC recovery in PCa surgery (16). Anatomical parameters such as membranous urethral length (MUL) (17), prostate volume (PV) (18), intravesical prostatic protrusion length (IPPL) (17), and prostatic apex morphology (19) have been investigated as potential predictors. Nevertheless, these metrics do not fully capture the functional anatomy that supports urethral closure and continence. The curvature of the male urethra, shaped by spatial relationships between the prostate, pubic bone, and surrounding supportive structures, serves a critical functional role in maintaining urinary continence (19). It enables coordinated action of the rhabdosphincter and puboperinealis muscles, which compress the urethra against the pubic bone under increased intra-abdominal pressure, facilitating urethral closure and preventing urinary leakage. Without this curvature, the urethra would be straight, hindering the generation of sufficient pressure to seal the urethra, thereby compromising the prevention of urine leakage (20). Although the functional importance of urethral curvature is recognized, prior studies have not quantified this parameter or validated its predictive performance for immediate UC after RARP.

We therefore hypothesized that urethral curvature reflects the functional continuity of the urethral support mechanism and may serve as an independent predictor of immediate UC recovery after RS-RARP. In this study, we aimed to introduce a novel mpMRI-based method for quantifying urethral curvature, validate its performance in preoperative assessment of immediate UC recovery following RS-RARP, and develop a multiparametric predictive nomogram combining urethral curvature with established anatomical predictors for clinical guidance in postoperative UC recovery.

## MATERIALS AND METHODS

### Study design and patients

This retrospective study was approved by the Ethics Committee of the First Affiliated Hospital University (approval number: MRCTA, ECFAH of FMU [2025] 002), and the study protocol was registered at clinicaltrials.gov (registration number: NCT06850116). Immediate UC was defined as patient-reported use of zero pads or one security liner per day within one week of catheter removal (21). Patients diagnosed with PCa who underwent RS-RARP at our center between January 2022 and January 2026 were included and were assigned to the training and internal validation cohorts according to the admission time. Patients admitted between January 2022, and January 2025 were assigned to the training cohort (n=348), whereas those admitted between February 2025 and January 2026 were assigned to the internal validation cohort (n=103). Patients treated at Nanjing Drum Tower Hospital, Qilu Hospital of Shandong University, and Baoji Central Hospital between February 2025, and January 2026 were assigned to the external validation cohort (n=129) (Figure-1).

**Figure 1:**
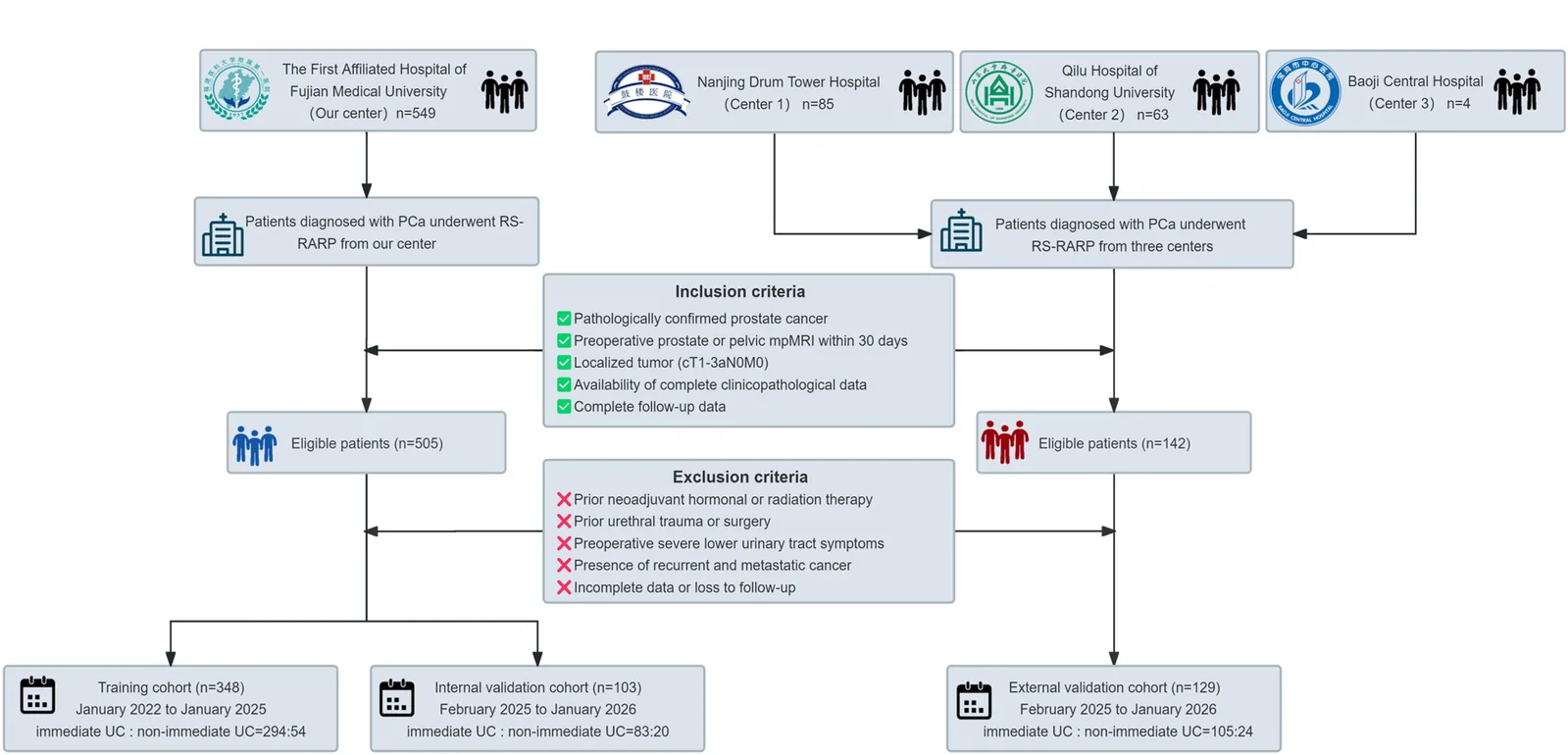
A flow diagram of the patient recruitment and study procedures.

The inclusion criteria were as follows: [1] prostate biopsy and postoperative pathology confirmed as prostate cancer; [2] mpMRI with a 3.0 T scanner for prostate or pelvic scans performed within 30 days before the operation; [3] localized tumor defined as cT1, cT2, or cT3aN0M0 in TNM staging, without regional lymph node metastasis or distant metastasis; [4] availability of complete clinicopathological data; [5] complete follow-up data. The exclusion criteria were as follows: [1] history of prior neoadjuvant treatments, including hormone therapy or radiotherapy; [2] history of previous urethral trauma or surgery; [3] severe lower urinary tract symptoms before operation; [4] presence of recurrent or metastatic cancer; [5] incomplete clinical, imaging, or pathological data, or loss to follow-up.

### MpMRI examination

The mpMRI imaging was performed using a magnetic resonance scanner (MAGNETOM™ Verio 3.0T, Siemens Healthineers) with a transabdominal external phased-array coil. Patients were positioned in a supine position with a moderately filled bladder during imaging. The following sequences were obtained: [1] axial, coronal, and sagittal T2-weighted imaging (T2WI) sequences; [2] axial T1-weighted imaging (T1WI) scans; [3] diffusion-weighted imaging (DWI); [4] apparent diffusion coefficient (ADC), and [5] dynamic contrast enhancement (DCE). The contrast agent (gadolinium Gd-DTPA) was injected through the cubital vein at a flow rate of 3.0 mL/s, and the contrast injection was simultaneously scanned for 15 cycles. The MRI image acquisition parameters are listed in Table-S1.

### MpMRI scanning evaluation

Preoperative anatomical measurements of mpMRI were evaluated with Prostate Imaging Reporting and Data System (PIRADS) version 2 (22). For each variable, measurements were independently conducted by two radiologists with ten (QJX) and eight (SBZ) years of experience, respectively, under the guidance of one experienced urologist (SHC). The measurers were blinded to each other's results, and both were blinded to the clinical information and follow-up outcomes of the patients. Discrepancies were resolved through consensus reading. All images were evaluated on T2WI sequences using the Philips MR workstation (IntelliSpace Portal, ISP). The measured mpMRI parameters were as follows:

MUL: the length of the urethra between the lower border of the prostatic apex and the upper border of the penile root (Figure-2A).

**Figure 2:**
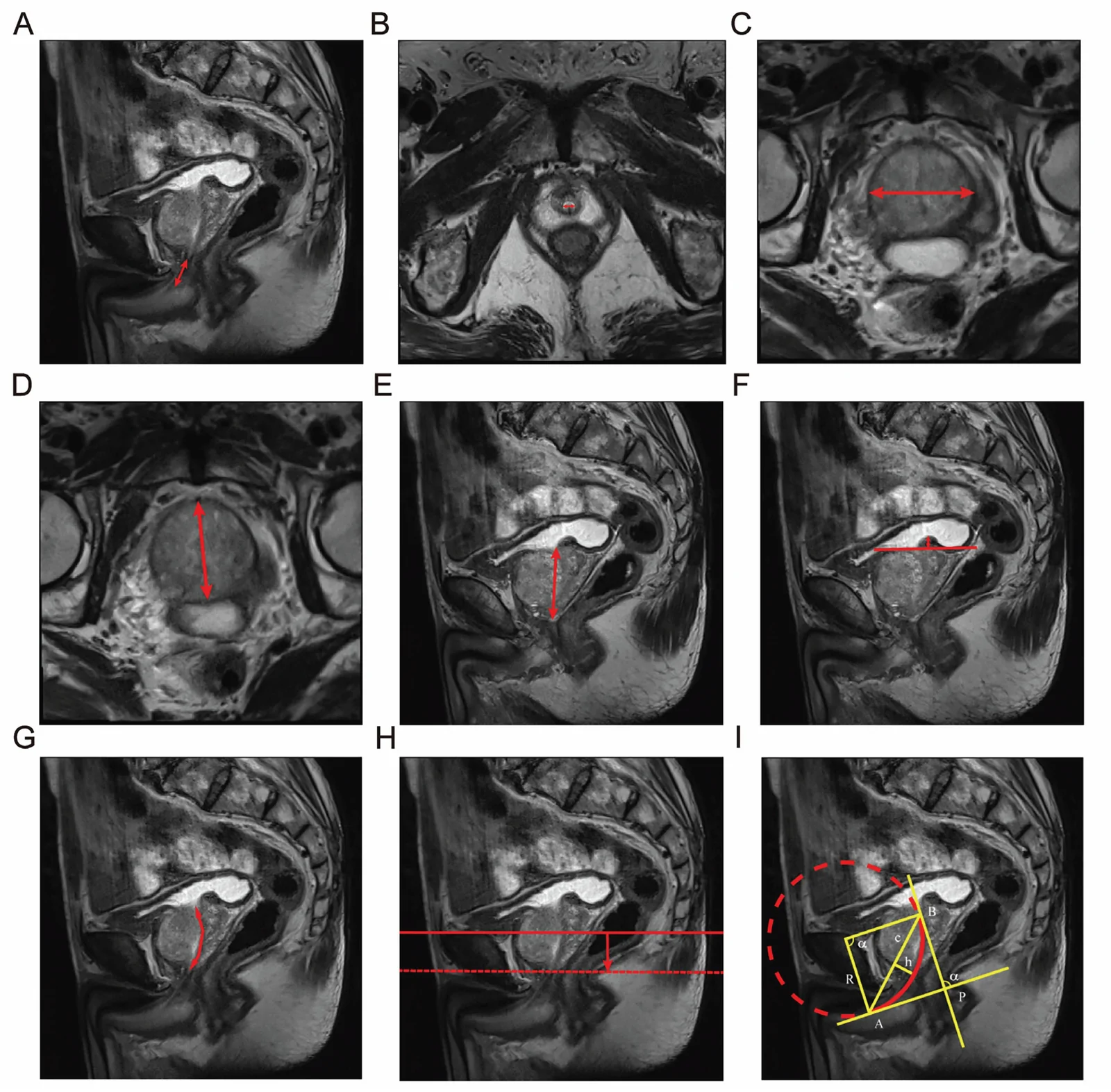
Prostate MRI parameters.

A) Membranous urethral length; B) Membranous urethral width; C) Prostate width; D) Prostate length; E) Prostate height; F) Intravesical prostatic protrusion length; G) Prostatic urethral length; H) Pubic symphysis-prostate apex length; I) Measurement of urethral curvature (solid red line). The chord length (c) was defined as the line connecting the bladder neck and the origin of the membranous urethra, whereas the height (h) was the perpendicular distance from the chord to the urethral curve margin. The radius (R) of the fitted circle (dashed red line) is shown in yellow, and curvature was calculated as 1/R.

Membranous urethral width (MUW): the maximum diameter of the membranous urethra measured on the axial plane (Figure-2B).

PV: prostate width × length × height × (π/6) (Figures 2C-E).

IPPL: the vertical distance from the tip of the protruding prostate to the base of the urinary bladder on the midsagittal image (Figure-2F).

Prostatic urethral length (PUL): the length from the prostatic apex to the entrance of the urethral bulb on the sagittal plane (Figure-2G).

Pubic symphysis-prostate apex length (PAL): the distance between the extension lines of the suprapubic ridge and the prostate apical line on the sagittal MRI (Figure-2H).

Urethral curvature: reciprocal of the urethral curve radius between the bladder neck and the origin of the membranous urethra (Figure-2I). The radius was measured in centimeters (cm).

Specifically, to measure urethral curvature, the sagittal T2-weighted image slice with the clearest visualization of the urethral arc between the bladder neck and the origin of the membranous urethra was selected. The distance between the inflection points on both sides of the dome was considered (denoted as line c in Figure-2I). The distance from the apex of the dome to line c was measured as a perpendicular line (denoted as *h* in Figure-2I). The radius (*R*) of the hypothetical circle defined by its chord c and height h was then calculated. The geometric formula used to determine R is as follows:


$$R=\frac{h}{2}+\frac{c^{2}}{8h}$$

The mean curvature K of the urethral curve segment is defined as the ratio of the total tangent turning angle α (in radians) to the arc length∩⌒AB (s); For an arbitrary arc∩ AB on the circle (Figure-2I), the tangent turning angle α equals the central angle subtended by the arc. By the circular arc length formula s=Rα, substituting into the mean curvature definition directly yields:


$$\text{K}=\frac{\alpha }{s}=\frac{1}{R}=\frac{1}{R}$$

The pointwise curvature K at any point on the circle is the limit of as the arc length approaches zero:


$$K=\underset{s\to 0}{\lim}\frac{1}{R}=\frac{1}{R}$$

Lee-type (19): A) The prostate apex covers the anterior and posterior parts of the membranous urethra (Figure-3A); B) the prostate apex covers the anterior part of the membranous urethra (Figure-3B); C) the prostate apex covers the posterior part of the membranous urethra (Figure-3C); D) the prostate apex does not cover the membranous urethra (Figure-3D).

**Figure 3:**
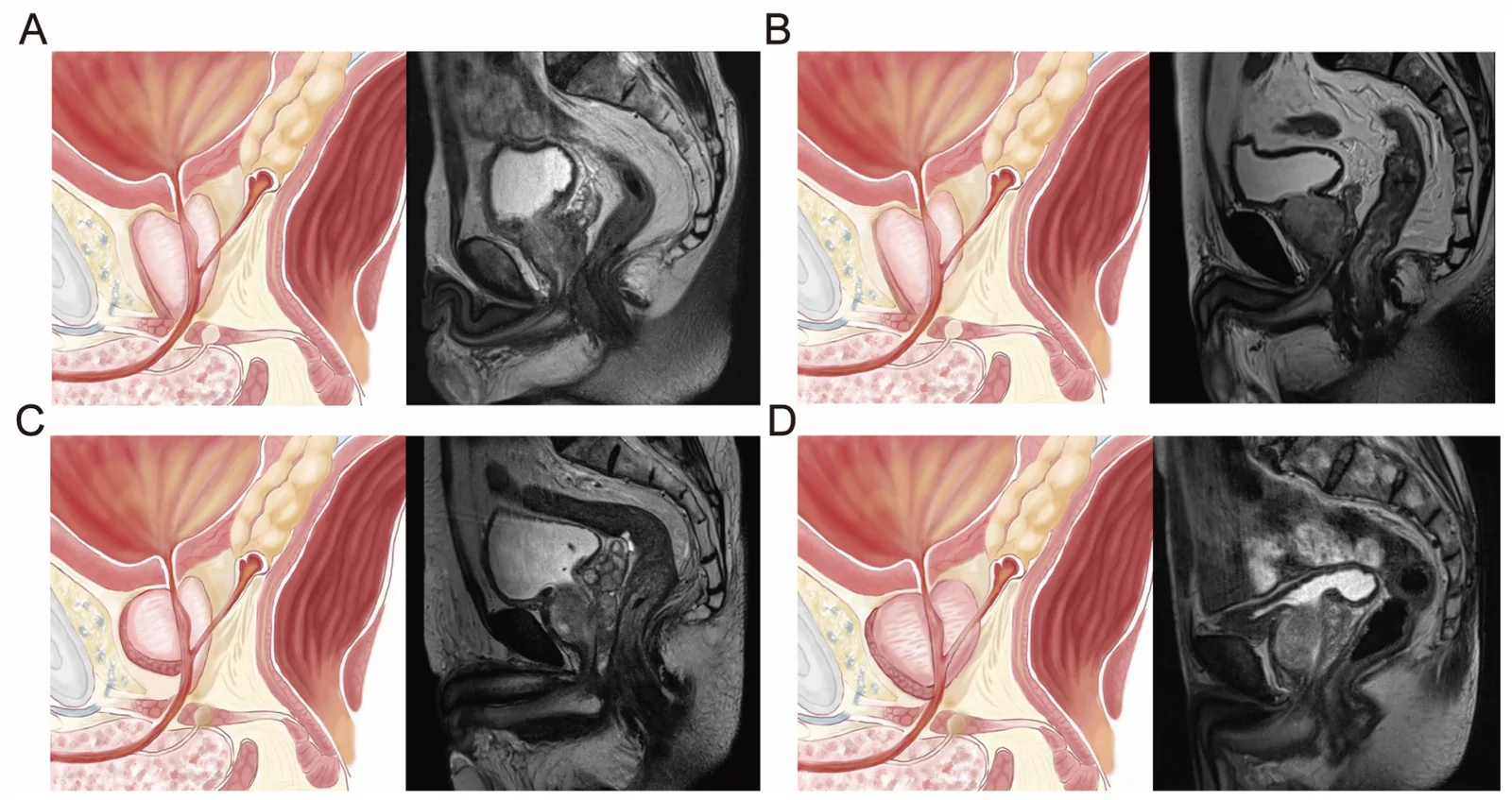
A schema illustrating the four types of prostatic apex.

A) Lee-type A: the prostate apex covers the anterior and posterior parts of the membranous urethra; B) Lee-type B: the prostate apex covers the anterior part of the membranous urethra; C) Lee-type C: the prostate apex covers the posterior part of the membranous urethra; D) Lee-type D: the prostate apex does not cover the membranous urethra

## SURGICAL TECHNIQUES

All procedures were performed by experienced surgeons with more than 5 years of experience in RS-RARP, each of whom performed more than 100 RS-RARP procedures annually. The procedure was performed using the Da Vinci Xi™ surgical system. The RS-RARP surgical approach was based on the techniques described by Lim et al. (23), bladder neck preservation method was based on the techniques described by Galfano et al. (12), the neurovascular bundle preservation method was based on the techniques described by Kowalczyk et al (24), and the urethral length preservation method was based on the techniques described by Hamada et al. (25).

### Data collection

Demographic and clinical features: age, body mass index (BMI), presence of hypertension, diabetes, and prostate surgery history, D’Amico risk classification, PIRADS score, international prostate symptom score (IPSS), and clinical TNM staging (NCCN guideline 2022.4).

Perioperative outcomes and pathological characteristics: preoperative prostate-specific antigen (PSA) level, operative time (from skin incision to closure), estimated blood loss, blood transfusion status, neurovascular bundle preservation, postoperative drainage volume, postoperative drain and catheter removal times, length of hospital stay, status of surgical margin and nerve invasion, and Gleason score of prostate biopsy.

MpMRI imaging measurements: including MUL, MUW, PV, urethral curvature, IPPL, PUL, PAL, and Lee-type.

## Statistical Analyses

Statistical analyses were performed using SPSS version 26.0 (IBM Corp, Armonk, NY, USA). Inter-observer agreement was assessed using weighted Cohen's kappa (26) for categorical variables and intraclass correlation coefficients (ICCs) (27) for continuous variables. Kappa values were interpreted as: poor (less than 0.20), fair (0.21-0.40), moderate (0.41-0.60), substantial (0.61-0.80) or almost perfect agreement (0.81-1.00). ICCs were interpreted as: poor (less than 0.40), fair (0.40-0.59), good (0.60-0.74), and excellent (0.75-1.00). Normally distributed data were expressed as mean ± standard deviation (SD) and analyzed using Student's t-test for comparisons between two groups or one-way ANOVA for comparisons among three or more groups. Non-normally distributed data were expressed as median and interquartile range (IQR) and analyzed using the Mann-Whitney U test for two-group comparisons or the Kruskal–Wallis test for three or more groups. Categorical variables were expressed as counts and percentages and analyzed using the chi-square or Fisher's exact test.

Univariate analysis and least absolute shrinkage and selection operator (LASSO) regression were used to screen for the best predictors. The selected predictors were then included in multivariate logistic regression analysis. Odds ratios (ORs) and 95% confidence intervals (CIs) were calculated to identify predictors of immediate UC after RS-RARP. The multivariate logistic regression results were presented as a nomogram, constructed using R software (R Foundation for Statistical Computing, Vienna, Austria, version 4.1.0). The models were internally validated using 1,000 bootstrap resampling iterations. Nomogram performance was evaluated using the AUC to assess discriminative ability. Calibration was assessed using bootstrap-corrected calibration curves with 1,000 bootstrap resamples, Brier scores, and mean squared error (MSE) to evaluate the agreement between predicted and observed probabilities. Bootstrap resampling was performed to reduce model optimism (28). Decision curve analysis was conducted to compare the clinical utility of the nomogram with individual parameters across the full range of risk thresholds (0-1). Pearson's coefficient and Variance Inflation Factor (VIF) were used to evaluate the correlation and the co-linearity of the final predictors (29). The predictive performance of the nomogram was assessed using ROC curve analysis with MedCalc version 15.2 (Ostend, Belgium), determining the optimal cutoff values, sensitivity, and specificity based on the maximum Youden index. The predictive performance of various parameters for immediate UC recovery following RS-RARP was assessed using positive predictive value (PPV) and negative predictive value (NPV). The area under the ROC curve (AUC) and the 95% CIs were calculated. The DeLong test was applied to compare the AUCs of different variables (30). A two-sided p-value < 0.05 was considered statistically significant.

## RESULTS

### Demographic, perioperative clinicopathological features and mpMRI imaging characteristics

A total of 580 patients with PCa who underwent RS-RARP were included in this study, comprising 451 cases from our center and 129 cases from external institutions, including Nanjing Drum Tower Hospital (n=78), Qilu Hospital of Shandong University (n=47), and Baoji Central Hospital (n=4). Among the 451 patients from our center, 348 were allocated to the training cohort and 103 to the internal validation cohort. The 129 patients from external institutions were assigned to the external validation cohort (Figure-1). Their demographic and clinical data are shown in Table-1. The median age was 68 years (range 62-74), and the median BMI was 24.09 kg/m2 (range 22.43-25.50). The clinical TNM staging revealed that 30.7% of patients were classified as T1, 62.2% as T2, and 7.1% as T3. Preoperative Gleason scores were 30.2% for 6, 48.6% for 7, and 21.2% for ≥ 8. The median preoperative PSA level was 11.12 ng/mL (range 7.49-18.68). Immediate UC recovery was observed in 83.1% (482/580) of all patients, which was 84.5% (294/348) in the training cohort, 81.4% (105/129) in the external validation cohort, and 80.6% (83/103) in the internal validation cohort.

**Table 1:** Baseline characteristics of patients.

		All patients (n=580)	Training cohort (n=348)	External validation cohort (n=129)	Internal validation cohort (n=103)
Median age, (y)		68.00 (62.00, 74.00)	67.00 (62.00, 74.00)	68.00 (63.00, 74.00)	69.00 (64.00, 76.00)
BMI, (kg/m2)		24.09 (22.43, 25.50)	24.08 (22.47, 25.27)	24.22 (22.29, 26.18)	24.08 (22.47, 25.13)
**Hypertension, (%)**					
	No	314 (54.1%)	200 (57.5%)	58 (45.0%)	56 (54.4%)
	Yes	266 (45.9%)	148 (42.5%)	71 (55.0%)	47 (45.6%)
**Diabetes, (%)**					
	No	347 (59.8%)	202 (58.0%)	85 (65.9%)	60 (58.3%)
	Yes	233 (40.2%)	146 (42.0%)	44 (34.1%)	43 (41.7%)
**D’Amico risk classification, (%)**					
	Low	42 (7.2%)	30 (8.6%)	5 (3.8%)	7 (6.8%)
	Intermediate	400 (69.0%)	246 (70.7%)	82 (63.6%)	72 (69.9%)
	High	138 (23.8%)	72 (20.7%)	42 (32.6%)	24 (23.3%)
**PIRADS score, (%)**					
	≤3	156 (26.9%)	89 (25.6%)	40 (31.0%)	27 (26.2%)
	>3	424 (73.1%)	259 (74.4%)	89 (69.0%)	76 (73.8%)
IPSS score		13.50 (7.00, 19.00)	13.00 (6.00, 19.00)	14.00 (10.00, 19.00)	14.00 (8.00, 20.00)
Preoperative PSA, (ng/mL)		11.12 (7.49, 18.68)	11.03 (7.49, 18.95)	9.79 (7.27, 16.14)	13.30 (8.39, 19.30)
**Clinical T stage, (%)**					
	T1	178 (30.7%)	111 (31.9%)	35 (27.1%)	32 (31.1%)
	T2	361 (62.2%)	217 (62.3%)	86 (66.7%)	58 (56.3%)
	T3	41 (7.1%)	20 (5.8%)	8 (6.2%)	13 (12.6%)
Operative time, (min)		115.28 (105.00,130.12)	116.60 (106.44, 130.80)	120.00 (100.00,130.00)	111.00 (106.00, 127.00)
Estimated blood loss, (mL)		60.00 (40.00, 80.00)	60.00 (50.00, 80.00)	80.00 (30.00, 90.00)	60.00 (30.00, 95.00)
Postoperative average drainage volume, (mL)		56.86 (30.28, 83.00)	57.36 (30.18, 82.00)	50.00 (25.00, 100.00)	62.00 (45.50, 79.00)
Postoperative drain removal time, (d)		3.00 (2.00, 4.00)	3.00 (2.00, 4.00)	3.00(2.00, 3.00)	3.00(2.00, 4.00)
Postoperative catheter removal time, (d)		15.00 (13.00, 20.00)	15.00 (13.00, 20.00)	15.00 (13.00, 18.00)	16.00 (14.00, 19.50)
Postoperative hospitalization duration, (d)		6.50 (5.00, 8.00)	7.00 (5.00, 8.00)	6.00 (5.00, 7.00)	7.00 (5.50, 8.00)
**NVB preservation, (%)**					
	No	317 (54.7%)	195 (56.0%)	70 (54.3%)	52 (50.5%)
	Unilateral	40 (6.9%)	19 (5.5%)	12 (9.3%)	9 (8.7%)
	Bilateral	223 (38.4%)	134 (38.5%)	47 (36.4%)	42 (40.8%)
**Intraoperative transfusion, (%)**					
	No	572 (98.6%)	345 (99.1%)	127 (98.5%)	100 (97.1%)
	Yes	8 (1.4%)	3 (0.9%)	2 (1.5%)	3 (2.9%)
**Surgical margin, (%)**					
	Negative	448 (77.2%)	260 (74.7%)	105 (81.4%)	83 (80.6%)
	Positive	132 (22.8%)	88 (25.3%)	24 (18.6%)	20 (19.4%)
Gleason score of prostate biopsy, (%)					
	6	175 (30.2%)	111 (31.9%)	32 (24.8%)	32 (31.1%)
	7	282 (48.6%)	163 (46.8%)	71 (55.0%)	48 (46.6%)
	≥8	123 (21.2%)	74 (21.3%)	26 (20.2%)	23 (22.3%)
**Nerve invasion, (%)**					
	No	358 (61.7%)	226 (64.9%)	68 (52.7%)	64 (62.1%)
	Yes	222 (38.3%)	122 (35.1%)	61 (47.3%)	39 (37.9%)
MUL, (mm)		15.61 (12.99, 18.06)	15.55 (12.96, 18.29)	15.44 (12.44, 17.95)	16.31 (13.63, 17.86)
MUW, (mm)		10.93 (9.55, 12.29)	10.82 (9.54, 12.20)	11.04 (9.84, 12.32)	11.19 (9.52, 12.51)
PV, (mL)		50.86 (44.87, 56.95)	50.56 (44.48, 56.39)	52.61 (46.67, 59.57)	50.09 (44.59, 56.14)
Urethral curvature, (cm-1)		0.316 (0.283, 0.348)	0.312 (0.286, 0.344)	0.322 (0.288, 0.352)	0.307 (0.271, 0.343)
**Lee-type, (%)**					
	A	133 (22.9%)	73 (21.0%)	38 (29.4%)	22 (21.4%)
	B	110 (19.0%)	66 (19.0%)	25 (19.4%)	19 (18.4%)
	C	45 (7.8%)	30 (8.6%)	5 (3.9%)	10 (9.7%)
	D	292 (50.3%)	179 (51.4%)	61 (47.3%)	52 (50.5%)
PUL, (mm)		37.25 ± 4.95	37.65 ± 5.04	36.50 ± 4.67	36.87 ± 4.92
IPPL, (mm)		4.35 (3.16, 5.45)	4.37 (3.10, 5.52)	4.64 (3.25, 5.52)	4.00 (3.12, 4.98)
PAL, (mm)		26.56 (24.47, 29.09)	26.39 (24.03, 29.10)	27.31 (25.44, 29.35)	26.02 (24.71, 28.87)
**Immediate UC, (%)**					
	No	98 (16.9%)	54 (15.5%)	24 (18.6%)	20 (19.4%)
	Yes	482 (83.1%)	294 (84.5%)	105 (81.4%)	83 (80.6%)

BMI = body mass index; PIRADS = prostate imaging reporting and data system; IPSS = international prostate symptom score; PSA = prostate specific antigen; NVB = neurovascular bundle; MUL = membranous urethral length; MUW = membranous urethral width; PV = prostate volume; PUL = prostatic urethral length; IPPL = Intravesical prostatic protrusion length; PAL = pubic symphysis-prostate apex length; UC = urinary continence.

### Comparison of the variables between the groups in the training cohort

The inter-observer reliability analysis demonstrated a strong correlation between the two observers for mpMRI imaging measurements, with ICCs ranging from 0.86 to 0.98 (Table-S2). Univariate analysis was performed to compare the variables between the immediate and non-immediate UC groups in the training cohort. The non-immediate UC group had a higher BMI (25.08 vs. 23.84 kg/m², P = 0.005), larger PV (58.64 vs. 49.34 mL, P < 0.001), and a higher proportion of T3 stages (13.0% vs. 4.4%, P = 0.046).

Bilateral NVB preservation, a key factor in preserving postoperative UC function, was significantly higher in the immediate UC group (41.8% vs. 20.4%, P < 0.001). Anatomical measurements showed significant differences between the groups. The immediate UC group had a longer MUL (16.00 vs. 12.83 mm, P < 0.001), shorter IPPL (4.27 vs. 4.96 mm, P = 0.028), shorter PAL (26.12 vs. 27.53 mm, P = 0.030), larger urethral curvature (0.321 vs. 0.285 cm-1, P < 0.001), and a larger proportion of Lee-type D (59.2% vs. 9.3%, P < 0.001) (Table-2).

**Table 2:** Baseline characteristics and perioperative outcomes of patients with immediate vs non-immediate urinary continence in the training cohort.

		immediate urinary continence (n=294)	non-immediate urinary continence (n=54)	P value
Median age, (y)		66.00 (61.25, 74.00)	69.00 (62.25, 75.00)	0.076a
BMI, (kg/m2)		23.84 (22.31, 25.03)	25.08 (23.10, 25.86)	0.005a
**Hypertension, (%)**				0.374b
	No	166 (56.5%)	34 (63.0%)	
	Yes	128 (43.5%)	20 (37.0%)	
**Diabetes, (%)**				0.090b
	No	165 (56.1%)	37 (68.5%)	
	Yes	129 (43.9%)	17 (31.5%)	
**D’Amico risk classification, (%)**				0.651b
	Low	26 (8.9%)	4 (7.4%)	
	Intermediate	205 (69.7%)	41 (75.9%)	
	High	63 (21.4%)	9 (16.7%)	
**PIRADS score, (%)**				0.457b
	≤3	73 (24.8%)	16 (29.6%)	
	>3	221 (75.2%)	38 (70.4%)	
IPSS score		13.00 (6.00, 19.75)	13.00 (3.00, 18.00)	0.194a
Preoperative PSA, (ng/mL)		10.48 (7.28, 18.70)	12.09 (8.87, 21.09)	0.113a
**Clinical T stage, (%)**				0.046b
	T1	95 (32.3%)	16 (29.6%)	
	T2	186 (63.3%)	31 (57.4%)	
	T3	13 (4.4%)	7 (13.0%)	
Operative time, (min)		116.37 (106.49, 129.91)	121.37 (105.19, 137.72)	0.158a
Estimated blood loss, (mL)		60.00 (50.00, 80.00)	60.00 (30.00, 70.00)	0.113a
Postoperative average drainage volume, (mL)		56.14 (28.00, 83.75)	63.24 (47.06, 79.16)	0.236a
Postoperative drain removal time, (d)		3.00 (2.00, 4.00)	3.00 (2.00, 4.00)	0.771a
Postoperative catheter removal time, (d)		15.00 (13.00, 20.00)	15.00 (12.00, 20.00)	0.919a
Postoperative hospitalization duration, (d)		7.00 (5.00, 8.00)	7.00 (5.00, 8.75)	0.273a
**NVB preservation, (%)**				<0.001b
	No	165 (56.1%)	30 (55.5%)	
	Unilateral	6 (2.1%)	13 (24.1%)	
	Bilateral	123 (41.8%)	11 (20.4%)	
**Intraoperative transfusion, (%)**				0.398b
	No	292 (99.3%)	53 (98.1%)	
	Yes	2 (0.7%)	1 (1.9%)	
**Surgical margin, (%)**				0.823b
	Negative	219 (74.5%)	41 (75.9%)	
	Positive	75 (25.5%)	13 (24.1%)	
**Gleason score of prostate biopsy, (%)**				0.620b
	6	94 (32.0%)	17 (31.5%)	
	7	137 (46.6%)	26 (48.1%)	
	≥8	63(21.4%)	11 (20.4%)	
**Nerve invasion, (%)**				0.116b
	No	196 (66.7%)	30 (55.6%)	
	Yes	98 (33.3%)	24 (44.4%)	
MUL, (mm)		16.00 (13.63, 18.51)	12.83 (11.26, 14.89)	<0.001a
MUW, (mm)		10.95 (9.66, 12.23)	10.33 (9.04, 11.53)	0.122a
PV, (mL)		49.34 (43.74, 53.89)	58.64 (51.10, 69.69)	<0.001a
Urethral curvature, (cm-1)		0.321 ± 0.044	0.285 ± 0.027	<0.001c
**Lee-type, (%)**				<0.001b
	A	59 (20.1%)	14 (25.9%)	
	B	38 (12.9%)	28 (51.8%)	
	C	23 (7.8%)	7 (13.0%)	
	D	174 (59.2%)	5 (9.3%)	
PUL, (mm)		37.55 ± 4.84	38.19 ± 6.02	0.392c
IPPL, (mm)		4.27 (3.08, 5.46)	4.96 (3.61, 6.25)	0.028a
PAL, (mm)		26.12 (23.84, 28.92)	27.53 (25.93, 29.87)	0.030a

a P values were calculated with Mann-Whitney U test; b P values were calculated with the chi-square test or Fisher's exact test; c P values were calculated with Student's t test.

BMI = body mass index; PIRADS = prostate imaging reporting and data system; IPSS = international prostate symptom score; PSA = prostate specific antigen; NVB = neurovascular bundle; MUL = membranous urethral length; MUW = membranous urethral width; PV = prostate volume; PUL = prostatic urethral length; IPPL = Intravesical prostatic protrusion length; PAL = pubic symphysis-prostate apex length.

### Logistic regression analysis identifying independent predictors of immediate urinary continence recovery after RS-RARP

Since the results of the univariate analysis could be influenced by confounding factors, the statistically significant indicators in Table-2 were selected as independent variables, and LASSO regression was performed to further screen variables with non-zero coefficients from the univariate logistic regression. The best predictors of immediate UC recovery following RS-RARP were NVB preservation, Lee-type D, clinical T stage, MUL, PV, and urethral curvature (Figures 4A and 4B).

**Figure 4:**
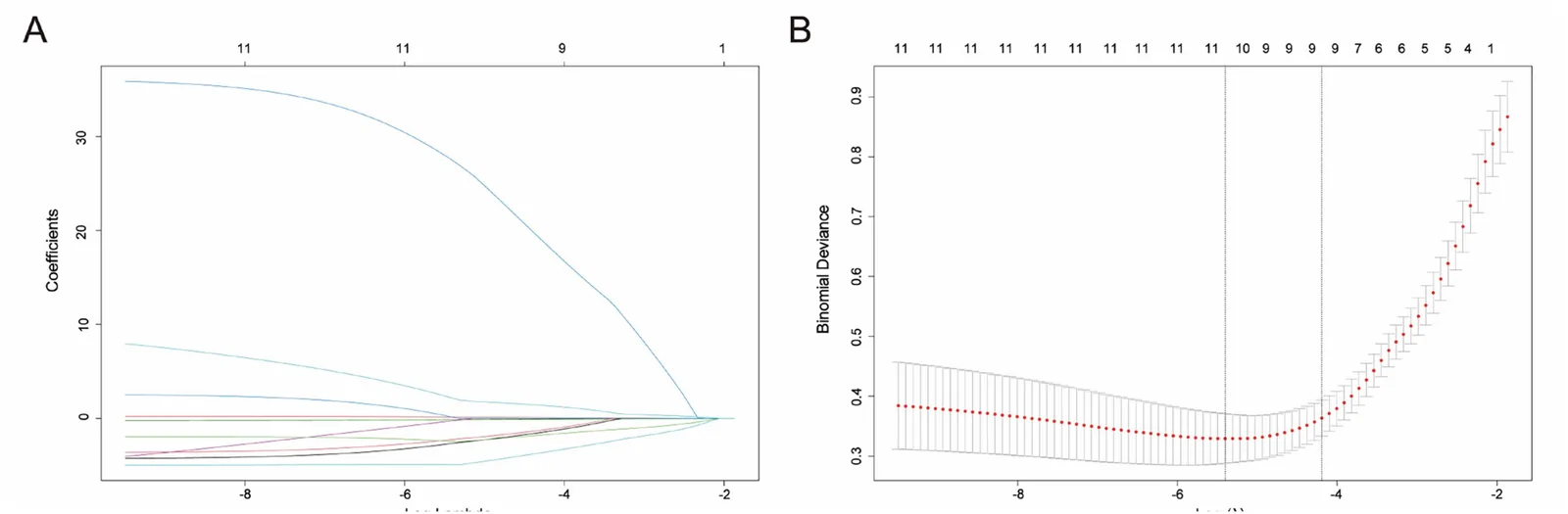
Selection of predictive factors using LASSO logistic regression algorithm.

A) LASSO coeficiente profiles for the 9 candidate variables; B) The optimal value was determined by the two dashed vertical lines, which were drawn based on the minimum mean square error criterion (left dashed line) and the standard error criterion (right dashed line). In this study, six predictors were selected according to the standard error criterion (λ = 0.015). LASSO, least absolute shrinkage and selection operator.

In further analyses, these six potential factors, along with clinically relevant parameters (including operative time) (31), were incorporated as confounders into the multivariable logistic regression model to identify reliable preoperative predictors of immediate UC recovery. The results demonstrated that MUL (OR=1.23, P=0.029) and urethral curvature (OR=2.84, P<0.001) were identified as independent predictors for immediate urinary continence recovery, while PV (OR=0.84, P<0.001) was identified as a protective factor (Table-3).

**Table 3:** Multivariate logistic regression analysis identifying independent predictors of immediate urinary continence recovery after RS-RARP and receiver operating characteristic analysis of their predictive performance.

Variable	Multivariate logistic regressionanalysis						Receiver operating characteristic analysis					
	B	Standard Error	P value	OR	95% CI		AUC	95% CI		Cutoff value	Sensitivity	Specificity
					Lower limit	Upper limit		Lower limit	Upper limit			
NVB preservation	-0.12	0.36	0.737	0.89	0.44	1.80						
Lee-type D	2.54	1.77	0.152	2.84	1.57	5.16						
MUL	0.20	0.09	0.029	1.23	1.02	1.47	0.74	0.69	0.79	14.98	0.80	0.64
PV	-0.18	0.04	<0.001	0.84	0.77	0.91	0.75	0.70	0.80	54.99	0.65	0.80
Urethral curvature	1.04	0.30	<0.001	2.84	1.57	5.16	0.76	0.71	0.80	0.323	1.00	0.48
Clinical T3 stage	-0.34	0.18	0.059	0.71	0.50	1.01						
Operative time	-0.06	0.22	0.775	0.94	0.61	1.45						

RS-RARP = Retzius-sparing robot-assisted radical prostatectomy; OR = odds ratio; CI = confidence interval; NVB = neurovascular bundle; MUL = membranous urethral length; PV = prostate volume; AUC = the area under the ROC curve.

### Construction and validation of a nomogram for predicting immediate urinary continence recovery following RS-RARP

In the training cohort, a nomogram was developed based on multivariate regression, incorporating MUL, PV, and urethral curvature, to predict immediate postoperative UC recovery after RS-RARP (Figure-5A). Pearson's coefficients were −0.13 between urethral curvature and PV, and 0.17 between urethral curvature and MUL. The VIF for both predictors was 1.014, indicating no multicollinearity between PV, MUL, and urethral curvature. ROC analysis showed that the AUC values for MUL, PV, and urethral curvature were 0.74, 0.75, and 0.76 respectively (Table-3 and Figure-5B). To improve clinical applicability, a multivariate prediction model was developed using a combination of the three parameters through permutation testing. The AUC values for the two-parameter combined tests (MUL+PV), (MUL+urethral curvature), and (PV+urethral curvature) were 0.83 (95% CI, 0.79-0.87), 0.82 (95% CI, 0.78-0.86), and 0.83 (95% CI, 0.79-0.87), respectively.

**Figure 5:**
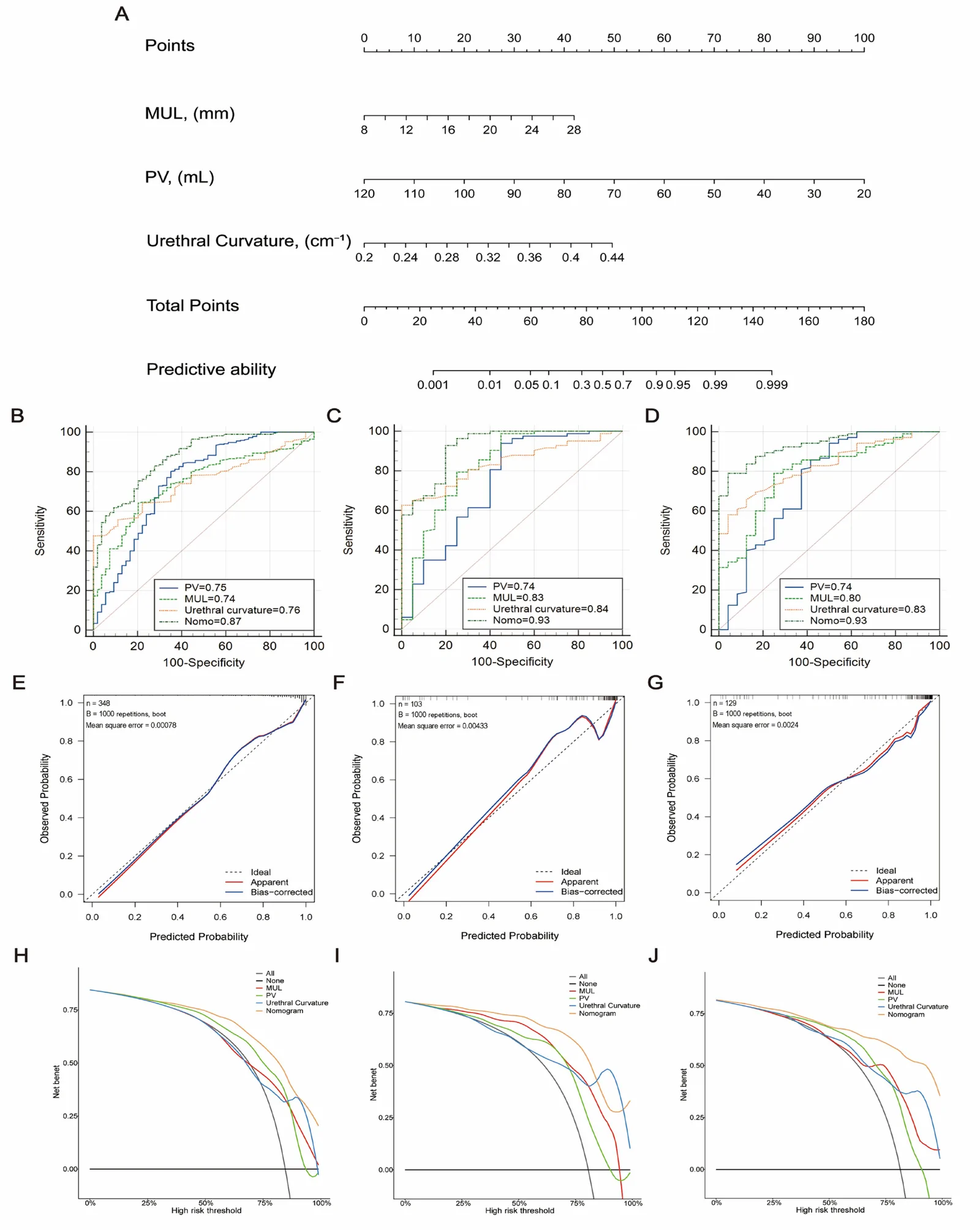
Construction and validation of a nomogram.

The nomogram model demonstrated robust discriminative performance, with an AUC of 0.87 (95% CI, 0.83-0.91; bootstrap-corrected, 0.87) in the training cohort (Figure-5B), surpassing those of the individual parameters and the two-parameter combined model, including MUL (DeLong test z = 4.05, P < 0.001), PV (z = 4.50, P < 0.001), urethral curvature (z = 3.96, P < 0.001), (MUL+PV) (z = 2.96, P = 0.003), (MUL+urethral curvature) (z = 2.01, P = 0.044), and (PV+urethral curvature) (z = 2.78, P = 0.005) (Table-S3). The nomogram demonstrated satisfactory discriminative performance, with an AUC of 0.93 (95% CI, 0.86-0.97; bootstrap-corrected, 0.92) in the internal validation cohort (Figure-5C) and an AUC of 0.93 (95% CI, 0.88-0.97; bootstrap-corrected, 0.92) in the external validation cohort (Figure-5D). The calibration curves demonstrated good agreement between the predicted probabilities and the observed immediate UC recovery rates. The bootstrap-corrected calibration slopes were 0.96, 0.84, and 0.89 for the training, internal validation, and external validation cohorts, respectively. The corresponding Brier scores were 0.08, 0.07, and 0.09, respectively. The corresponding mean squared error (MSE) values were 0.00078, 0.00433, and 0.00240, respectively, indicating good calibration in all three cohorts (Figures 5E-G). As illustrated in Figures 5H-J, decision curve analysis indicated that the nomogram provided a higher net benefit than the individual parameters across a broad range of reasonable threshold probabilities, demonstrating superior clinical utility for guiding decision-making.

## DISCUSSION

To our knowledge, this is the first multicenter study exploring the role of male physiological urethral curvature in urinary continence following RARP, including 580 patients (training cohort: n=348; internal validation cohort: n=103; external validation cohort: n =129). We present an innovative quantitative method for assessing urethral curvature and validate its reliability in predicting immediate UC recovery after RS-RARP using multicenter data (AUC of 0.76, 0.84, and 0.83 in the training, internal validation, and external validation cohorts, respectively). Furthermore, an mpMRI-based predictive model incorporating MUL, PV, and urethral curvature demonstrated excellent accuracy (AUC of 0.87, 0.93, and 0.93 in the training, internal validation, and external validation cohorts, respectively), outperforming individual parameters and dual-marker combinations (P < 0.05 for all DeLong tests). The bootstrap-corrected calibration curves and low MSE values confirmed the predictive accuracy of the nomogram. The excellent agreement between the predicted and observed probabilities following bootstrap resampling further supported the robustness of the proposed model. In addition, decision curve analysis demonstrated significantly improved net benefits across a range of threshold probabilities. This model offers a precise tool for assessing postoperative urinary incontinence risk and provides insights for developing individualized treatment strategies.

Preoperative mpMRI is crucial in diagnosing PCa and providing valuable anatomical data for both intraoperative and postoperative settings (32, 33). Chierigo et al. (34) highlighted that MRI-derived anatomical characteristics, particularly MUL, bladder neck position, and pelvic floor-related anatomical preservation, may be closely associated with early functional recovery after RS-RARP. MUL, the length of the urethra between the prostatic apex and the penile root, includes both smooth and striated muscle. During RARP, significant portions of striated muscle fibers surrounding the prostate are excised. Studies have shown that patients with a longer preoperative MUL tend to have a relatively longer membranous urethra postoperatively, which may facilitate earlier recovery of UC (35). Consistent with these observations, MUL was identified as an independent predictor in our cohort. Fonseca et al. (36) found that PV was linked to urinary incontinence at 3 and 6 months after RS-RARP, but not at 12 months, suggesting minimal impact on long-term UC recovery. However, our study identified smaller prostates (<53.94 mL) were associated with earlier recovery, likely due to larger prostates causing a larger bladder neck post-surgery, which increases surgical difficulty during bladder neck anastomosis. Prostatic enlargement may also lead to short-term bladder decompensation, contributing to postoperative urinary incontinence (18). Notably, our study demonstrated that preoperative urethral curvature was also independently associated with immediate UC recovery. This finding suggests that the preoperative geometric configuration of the urethra may provide additional prognostic information for individualized risk stratification.

The intra-abdominal curvature of the male urethra is crucial for maintaining urinary continence by increasing urethral pressure and enhancing sphincter function (37). Zhang et al. (38) demonstrated that an appropriately curved urethra increases urethral pressure, aiding continence. Furthermore, this curvature modifies urine flow dynamics and enhances sphincter control, which is crucial under increased abdominal pressure, thereby reducing the risk of incontinence. Wang et al. (39) used computational fluid dynamics simulations to demonstrate that urethral curvature enhances the urethra's adaptability to pressure changes, thereby reinforcing continence. These findings highlight the critical role of urethral curvature in preventing incontinence by optimizing pressure distribution and flow dynamics. In light of this, surgical procedures such as RS-RARP with a posterior approach aim to preserve the natural urethral curvature by maintaining urethral support structures, including the pubovesical ligaments and bladder neck, ensuring optimal curvature through anastomosis between the bladder neck and urethral stump. In contrast, conventional RARP with an anterior approach may disrupt these structures, potentially leading to urethral elongation or straightening, which can negatively impact continence (40). This likely explains the difference in immediate urinary continence rates between RS-RARP and conventional RARP (41). To quantify this physiological urethral curvature, we introduce urethral curvature, the reciprocal of the urethral curve radius between the bladder neck and the origin of the membranous urethra. It provides an objective and repeatable measure to urethral curve and offers new insights into urethral morphology. Our findings suggest that the urethral curvature is associated with the immediate UC recovery following RS-RARP, with an optimal cutoff value of 0.323 cm-1 in the training cohort.

Furthermore, previous studies have suggested that the shape of the prostate apex influences immediate urinary continence recovery after RS-RARP (42). In this study, we classified prostate apex shape according to the Lee et al. classification (19). Our results showed that patients with Lee-type D had higher rates of immediate urinary continence, consistent with findings by Wenzel et al. (42) and Lacovelli et al. (43). This may be attributed to the higher position of the prostate apex, which spares the membranous urethra, facilitating better preservation of its integrity and function during surgery, thereby minimizing urethral trauma and enhancing urinary continence recovery. Beyond anatomical predictors, patient-related clinical characteristics may also contribute to UC recovery after RS-RARP. Dell’Oglio et al. (44) reported that the probability of immediate and 12-month UC recovery progressively declined with increasing age at surgery. Although age was not identified as an independent predictor in our cohort, this discrepancy may be related to differences in patient characteristics, sample size, and model construction. Further studies are warranted to clarify the combined contributions of anatomical and clinical factors to UC recovery.

From a clinical practice perspective, the measurement of urethral curvature and the construction of the predictive nomogram show excellent feasibility. All parameters are derived from standard preoperative 3.0 T mpMRI (T2WI sequences) without requiring additional imaging sequences or prolonged scan time. Furthermore, the reliance on standard anatomical landmarks and simple geometric calculations facilitates straightforward implementation in routine radiological practice with minimal additional training, while the inter-observer agreement (ICC: 0.86-0.98) confirms its high reproducibility. With ongoing advances in artificial intelligence-assisted image segmentation and quantitative image analysis, automated or semi-automated measurement tools may further improve measurement efficiency and reproducibility, thereby facilitating broader clinical implementation (45). The nomogram provides a clear, visual, and instantaneous risk stratification tool that can be used during preoperative counseling and surgical planning without complex computational support. Consequently, this method can be easily integrated into routine clinical pathways for patients undergoing RS-RARP.

Despite these noteworthy findings, our study has several limitations. First, the retrospective design may introduce potential selection bias. In addition, the exclusion of patients with severe preoperative LUTS may limit the generalizability of our findings. Further prospective studies are needed to verify the conclusions. Second, although mpMRI was analyzed by experienced urological imaging specialists under the supervision of an experienced urologist, interobserver variability cannot be completely excluded. Finally, due to the short follow-up period, long-term urinary continence rates and their influencing factors were not analyzed. Further studies with longer follow-up are warranted to validate the predictive value of this model for long-term continence recovery.

## CONCLUSION

Our study proposes a novel quantitative method for assessing urethral curvature and validates its reliability in predicting immediate UC recovery following RS-RARP. This method is simple, reproducible, and highly feasible for implementation in routine clinical practice. Additionally, by utilizing mpMRI-based indicators, a nomogram was constructed and validated in both internal and external cohorts, demonstrating superior predictive accuracy, good calibration, and favorable clinical utility. This model provides a valuable quantitative tool for preoperative risk stratification and personalized surgical planning.

## Data Availability

The data presented in the study are included in the article, and further inquiries can be directed to the corresponding authors.

## ABBREVIATIONS

RS-RARP: Retzius-Sparing Robot-Assisted Radical Prostatectomy
RARP: Robot-Assisted Radical Prostatectomy
RP: Radical Prostatectomy
PCa: Prostate Cancer
mpMRI: Multiparametric Magnetic Resonance Imaging
T2WI: T2-Weighted Imaging
T1WI: T1-Weighted Imaging
DWI: Diffusion-Weighted Imaging
PIRADS: Prostate Imaging Reporting and Data System
UC: Urinary Continence
MUL: Membranous Urethral Length
MUW: Membranous Urethral Width
PV: Prostate Volume
IPPL: Intravesical Prostatic Protrusion Length
PUL: Prostatic Urethral Length
PAL: Pubic Symphysis-Prostate Apex Length
ROC: Receiver Operating Characteristic
AUC: Area Under the ROC Curve
CI: Confidence Interval
OR: Odds Ratio
LASSO: Least Absolute Shrinkage and Selection Operator
MSE: Mean Squared Errors
VIF: Variance Inflation Factor
BMI: Body Mass Index
PSA: Prostate-Specific Antigen
IPSS: International Prostate Symptom Score
NVB: Neurovascular Bundle
ICC: Intraclass Correlation Coefficient
PPV: Positive Predictive Value
NPV: Negative Predictive Value
