# Supplementary material for: Could the preoperative urethral curve be used to predict immediate urinary continence following Retzius-sparing robot-assisted radical prostatectomy? A retrospective multicenter study
Source: Int Braz J Urol. 2026 Aug 5;52(6):e20260376. doi: 10.1590/S1677-5538.IBJU.2026.0376 (PMC13682737; doi:10.1590/S1677-5538.IBJU.2026.0376)
Supplement: Supplementary file 1 [file 1677-6119-ibju-52-6-e20260376-Suppl01.pdf]

Table S1 - Sequence parameters for mpMRI.

Parameters	T2WI	T1WI	DWI	DCE
TR/TE, ms	5960/93	573/11	6900/93	5.1/1.7
Flip angle, °	150	150	N/A	15
Echo train length	25	3	N/A	N/A
FOV, mm2	160×160	320×320	190×260	260×260
Matrix size	256×256	256×192	102×160	138×192
Slice thickness, mm	3.0	5.0	3.0	3.6
Others			b values = 0, 50, 800, 1500 sec/mm2	Temporal resolution = 9.3 s, total scan time of 3.53 min

mpMRI = multiparametric magnetic resonance imaging; T2WI = T2-weighted imaging; T1WI = T1-weighted imaging; DWI = diffusion-weighted imaging; ADC = apparent diffusion coefficient; DCE = dynamic contrast enhancement; 3D-GRE = three dimensional-gradient echo; TR = repetition time; TE = time echo; FOV = field of view; N/A = not available.

Table S2 - Inter-observer agreement in mpMRI parameter measurements for individual radiologists.

mpMRI imaging measurements	ICCa or Cohen's kappab		
	All patients (n=580)	immediate urinary continence (n=482)	non-immediate urinary continence (n=98)
MUL	0.97(0.96-0.98)	0.96(0.95-0.97)	0.98(0.97-0.99)
MUW	0.97(0.96-0.98)	0.97(0.96-0.98)	0.98(0.91-0.99)
PV	0.96(0.93-0.97)	0.95(0.92-0.97)	0.95(0.87-0.98)
Urethral curvature	0.89(0.78-0.94)	0.88(0.75-0.93)	0.86(0.74-0.92)
PUL	0.95(0.94-0.96)	0.95(0.94-0.96)	0.96(0.91-0.98)
IPPL	0.96(0.95-0.97)	0.96(0.94-0.97)	0.98(0.96-0.99)
PAL	0.90(0.88-0.91)	0.90(0.88-0.91)	0.90(0.86-0.93)
Lee-type	0.84(0.80-0.88)	0.82(0.77-0.86)	0.80(0.70-0.90)

aICC was used for continuous variables (e.g., MUL, MUW, PV, urethral curvature, PUL, IPPL, PAL).

bCohen's kappa was used for categorical variables (e.g., Lee-type).

ICC = Intraclass correlation coefficient; mpMRI = multiparametric magnetic resonance imaging; MUL = membranous urethral length; MUW = membranous urethral width; PV = prostate volume; PUL = prostatic urethral length; IPPL = Intravesical prostatic protrusion length; PAL = pubic symphysis-prostate apex length.

Table S3 - Delong test comparing different ROC curves in the training cohort.

Comparison	AUC difference	Standard Error	Z value	P value	95% CI	
					Lower limit	Upper limit
MUL~nomogram	0.13	0.03	4.05	<0.001	0.07	0.19
PV~nomogram	0.12	0.03	4.50	<0.001	0.07	0.17
Urethral curvature ~nomogram	0.11	0.03	3.96	<0.001	0.06	0.18
MUL+PV~nomogram	0.04	0.01	2.96	0.003	0.01	0.07
MUL+Urethral curvature~nomogram	0.05	0.02	2.01	0.044	0.01	0.10
PV+Urethral curvature~nomogram	0.04	0.01	2.78	0.005	0.01	0.07

ROC, = receiver operating characteristic; AUC = the area under the ROC curve; CI = confidence interval; MUL = membranous urethral length; PV = prostate volume.
